# Maladie de Sneddon-Wilkinson: à propos d’un cas

**DOI:** 10.11604/pamj.2022.43.115.33116

**Published:** 2022-11-01

**Authors:** Sara Kerroum, Najoua Ammar, Kaoutar Znati, Nadia Ismaili, Mariame Meziane, Laila Benzekri, Karima Senouci

**Affiliations:** 1 Centre Hospitalier Universitaire Ibn Sina, Service de Dermatologie-Vénérologie, Faculté de Médecine et de Pharmacie, Université Mohamed V, Rabat, Maroc,; 2 Centre Hospitalier Universitaire Ibn Sina, Service d´Anatomo-Pathologie, Faculté de Médecine et de Pharmacie, Université Mohamed V, Rabat, Maroc

**Keywords:** Pustulose sous-cornée de Sneddon-Wilkinson, dapsone, cas clinique, Subcorneal pustulosis (Sneddon-Wilkinson disease), dapsone, case report

## Abstract

La maladie de Sneddon-Wilkinson est une pustulose amicrobienne, bénigne appartenant au spectre des dermatoses neutrophiliques. Sa présentation clinique est très stéréotypée et dans la plupart des cas, il s´agit de lésions pustuleuses du tronc et des grands plis. Cette pustulose peut être associée à d´autres pathologies (gammapathie monoclonale à IgA, polyarthrite rhumatoïde, néoplasies ou d´autres dermatoses neutrophiliques…) et donc nécessite un suivi régulier. Elle évolue de façon chronique par poussées- rémissions. Le traitement de première intention est la dapsone. Nous rapportons dans cet article le cas d´un patient âgé de 49 ans présentant une pustulose amicrobienne de Sneddon-Wilkinson.

## Abstract (en)

Sneddon-Wilkinson disease is a benign amicrobial pustulosis belonging to the spectrum of neutrophilic dermatoses. It is characterized by evident stereotypic clinical features, including, in most cases, pustular lesions of the trunk and prominent skin folds. This pustulosis may be associated with other diseases (IgA monoclonal gammopathy, rheumatoid arthritis, neoplasms or other neutrophilic dermatoses) and therefore requires regular follow-up. This disease has a relapsing-remitting course. First line therapy is dapsone. We here report the case of a 49-year-old patient with amicrobial pustulosis (Sneddon-Wilkinson disease).

## Introduction

La maladie de Sneddon-Wilkinson est une dermatose rare qui appartient au spectre des dermatoses neutrophiliques [1]. La forme classique touche habituellement les femmes entre 40 et 60 ans, sa présentation clinique est assez stéréotypée. Il s´agit d´une dermatose bénigne mais chronique et pouvant être associée à d´autres pathologies, justifiant une surveillance régulière et prolongée. Nous rapportons un cas de pustulose amicrobienne de Sneddon-Wilkinson.

## Patient et observation

**Figure 1:**
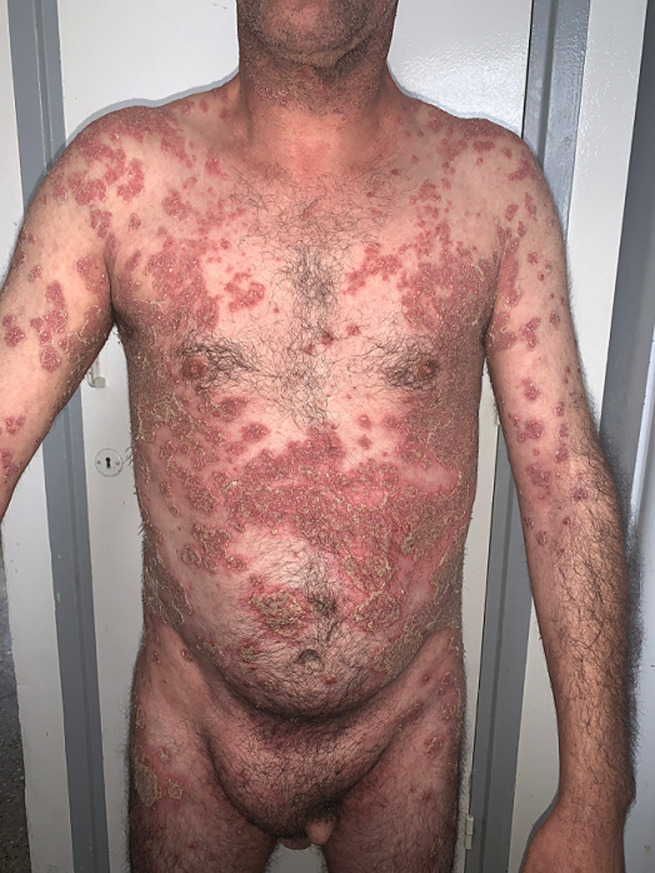
éruption érythémateuse pustuleuse du tronc et des membres supérieurs

**Figure 2:**
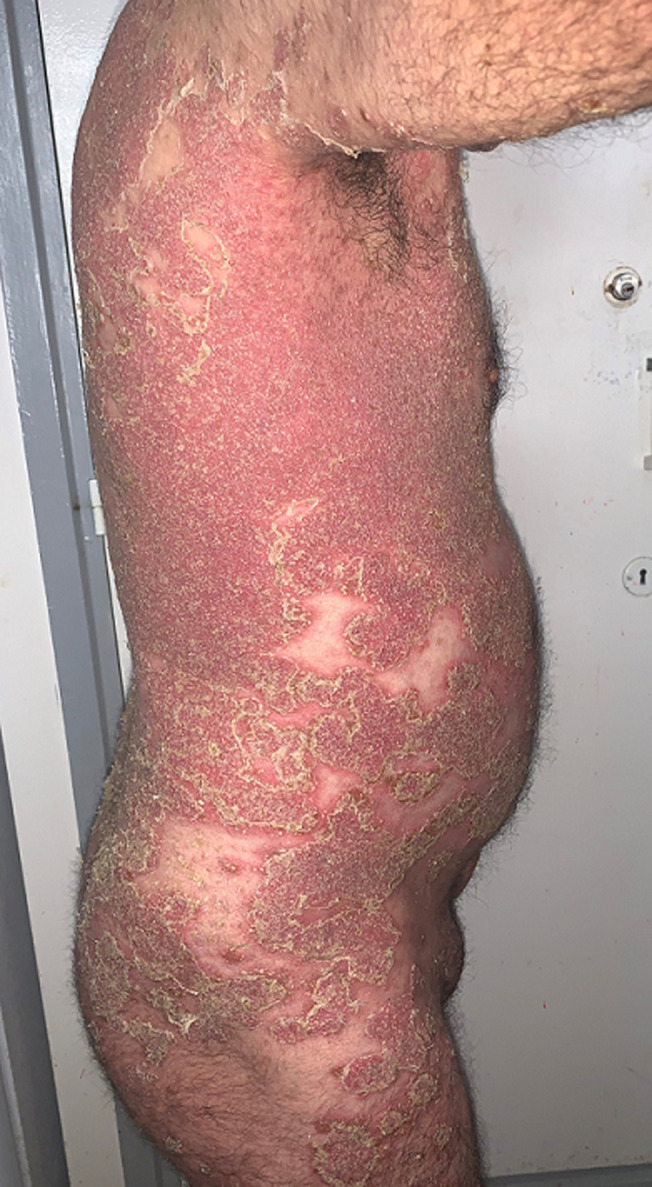
atteinte des plis et des membres inférieurs

**Informations relatives aux patients (présentation du patient):** nous rapportons le cas d´un patient âgé de 49 ans, ayant comme antécédent une hernie inguinale non compliquée qui consultait pour une éruption érythémateuse pustuleuse localisée au tronc, dos, aux plis axillaires et inguinaux et aux membres, évoluant depuis plusieurs mois par poussée- rémission (Figure 1, Figure 2).

**Résultats cliniques:** l´examen clinique a révélé de multiples pustules à hypopion parfois isolées ou en bordures de plaques érythémateuses à contours polycycliques. Cette éruption épargnait le visage, les zones palmo-plantaires ainsi que les muqueuses.

**Figure 3:**
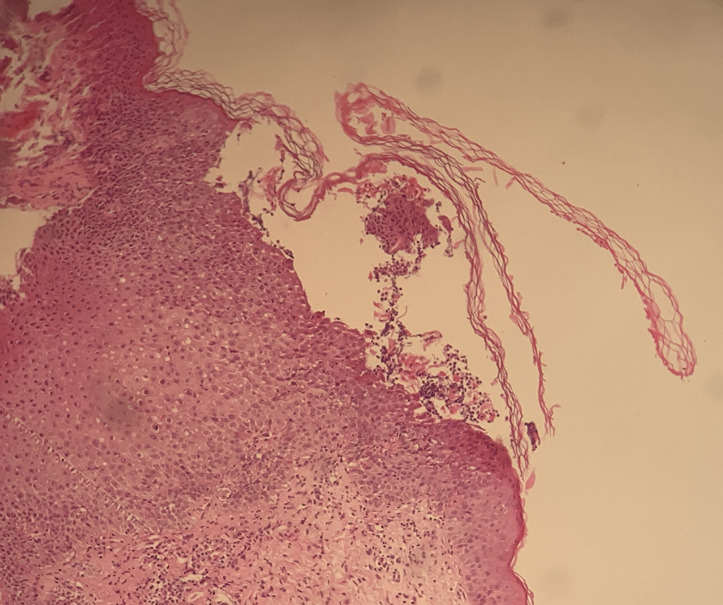
aspect histologique montrant une pustule sous-cornée

**Démarche diagnostique:** l´examen histologique montrait des pustules sous-cornées avec des amas de polynucléaires neutrophiles sans acantholyse (Figure 3). L´immunofluorescence directe, les prélèvements bactériologiques et mycologiques réalisés étaient négatifs. Le diagnostic de pustulose de Sneddon-Wilkinson a été retenu devant l´ensemble des éléments cliniques et histologiques. Des examens clinique et paraclinique approfondis ne montraient pas d´association pathologique chez ce patient.

**Intervention thérapeutique:** le patient a été traité initialement par une corticothérapie orale à raison de 1,5 mg/kg/j sans nette amélioration clinique d´où l´adjonction de la dapsone à la posologie de 2 mg/kg/j.

**Figure 4:**
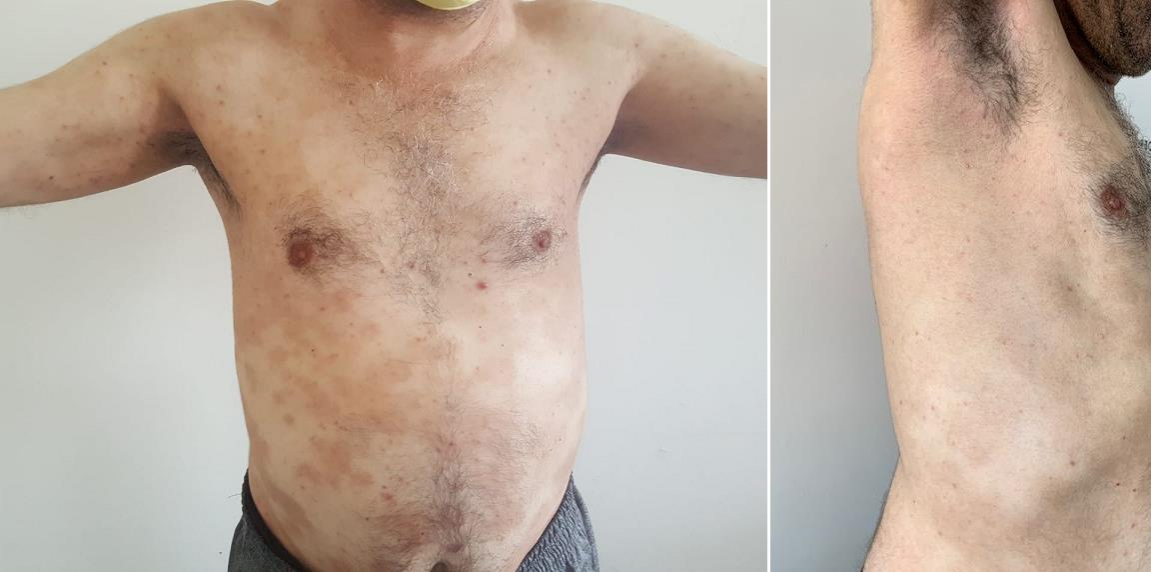
amélioration clinique après traitement

**Suivi et résultats des interventions thérapeutiques:** on constate une nette et rapide amélioration après l´introduction de la dapsone avec une desquamation des pustules (Figure 4).

**Perspectives du patient:** durant toute la période de traitement le patient était satisfait des soins reçus et optimiste quant à l´évolution thérapeutique.

**Consentement éclairé:** le patient a été informé du rapport de cas, des raisons pour lesquelles son cas était particulier et de l'intérêt des auteurs à publier son cas. Il a volontairement donné son consentement éclairé pour permettre aux auteurs d'utiliser ses photos pour ce rapport de cas.

## Discussion

La maladie de Sneddon-Wilkinson est une dermatose relativement rare, appartenant au spectre des dermatoses neutrophiliques. Elle touche l´adulte d´âge moyen avec une plus grande fréquence chez les sujets de sexe féminin [1], ce qui ne correspondait pas à notre patient qui lui était de sexe masculin. Sur le plan clinique, elle se caractérise par une éruption vésiculo-pustuleuse évoluant par poussées, constituée de pustules flasques de grande taille à hypopion confluant pour former des placards annulaires ou polycycliques [2]. Elle siège préférentiellement sur le tronc, les plis et les zones de flexion, épargnant classiquement les zones palmo-plantaires, le visage et les muqueuses. L´état général est bien conservé et il est rare que les poussées soient accompagnées de fièvre. Les signes fonctionnels se limitent à un prurit discret. L´examen histologique est indispensable au diagnostic et permet d´éliminer les autres dermatoses pustuleuses. Il montre de façon caractéristique une pustule amicrobienne intra-épidermique uniloculaire, sous-cornée, remplie de polynucléaires, surmontant un épiderme non modifié. Cet aspect histologique évocateur a été retrouvé chez notre patient. Les examens en immunofluorescence direct et indirect sont négatifs dans la grande majorité des cas. Il n´y a pas d´immunomarquage spécifique. Plusieurs associations pathologiques ont été rapportées comme: les gammapathies monoclonales, les autres dermatoses neutrophiliques, la polyarthrite rhumatoïde, les maladies inflammatoires chroniques de l´intestin, la maladie de Basedow, le lupus érythémateux disséminé…[3]. Notre patient ne présentait aucune pathologie associée.

Le traitement de première intention est la dapsone à la dose quotidienne de 50 à 150 mg, en cas de mauvaise tolérance hématologique ou d´échec, d´autres traitements ont montré leur efficacité inconstante: la colchicine, les rétinoides, les corticostéroides oraux, la photothérapie, le méthotrexate, et plus récemment les anti-TNF alpha [4]. Notre patient a bien répondu à la dapsone avec une nette amélioration clinique.

## Conclusion

La pustulose amicrobienne de Sneddon-Wilkinson a été peu décrite et est dans certains cas associée à d´autres pathologies, d´où l´importance d´un suivi régulier des patients.
